# Self-Concept Profiles in Lower Secondary Level – An Explanation for Gender Differences in Science Course Selection?

**DOI:** 10.3389/fpsyg.2019.00836

**Published:** 2019-04-24

**Authors:** Steffani Saß, Nele Kampa

**Affiliations:** Leibniz Institute for Science and Mathematics Education (IPN), Kiel, Germany

**Keywords:** self-concept profiles, dimensional comparison theory, person-centered approach, gender differences, course choice

## Abstract

One of the most powerful determinants of course selection in upper secondary level is undoubtedly students’ self-concept. Students with a high self-concept in a domain are more likely to select a course in that domain. However, according to the dimensional comparison theory, the formation of self-concept includes comparison processes with self-concepts in other domains. Regarding gender, females are less likely to choose physics and are more likely to have lower STEM self-concepts as well as lower aspirations toward STEM careers than males. In Germany, students in Grade 10 choose specific academic tracks to attend during upper secondary school. The academic track choice goes in hand with choosing advanced courses. This choice entails the decision about whether to pursue STEM subjects. We adopted the person-centered approach of latent profile analysis (LPA) to investigate the patterns of students’ self-concepts across the five domains, math, biology, reading, English, and physics. Furthermore, we investigated how those patterns influence educational choices regarding science subjects in upper secondary school in Germany. Based on a sample of 1,658 students, we tested whether the distinct profiles of self-concept in different domains in Grade 8 predicted gendered science course selection in Grade 10 as well as career aspirations in science. LPAs yielded four distinct profiles of self-concept that differed in level and shape: high math, high verbal, low overall, and high overall. These profiles were equivalent across gender. Gender differences were manifested in the relative distribution across the four profiles: females were more present in the low overall and high verbal-related self-concept profiles and males in the overall high and high math-related self-concept profiles. The profiles differed regarding abilities, choice of science course in upper secondary level, and science career aspirations.

## Introduction

Despite women increasingly entering higher education and occupations in general, they are still underrepresented in STEM occupations and university attendance (additional material of National Science Foundation, 2003, 2015a,b; Organisation for Economic Co-operation and Development [OECD], 2006, 2016). Field of study choices differ between males and females (e.g., Trusty, 2002; Nagy et al., 2006; Perez-Felkner et al., 2017) and are already channeled through course choice patterns in upper secondary education. Previous studies have found that, while females already opt out of STEM subjects during school, males more frequently choose advanced science-related or math subjects (Pinxten et al., 2012). Consequently, these first decisions in favor of or against STEM courses in secondary school influence future long-term educational aspirations such as the decision for STEM studies and occupations (Trusty, 2002; Ayalon, 2003; Lörz et al., 2011). One of the most prevalent predictors for opting out of STEM subjects during school is domain-specific self-concept (Wang et al., 2013; Guo et al., 2015). However, most research on course selection focuses on the unique effects of self-concept in a particular domain on course choice in the respective domain. Only few studies have investigated the effects of a combination of different academic self-concepts on course selection (Nagy et al., 2006; Marsh et al., 2009; Guo et al., 2017). These studies found that students compare their perceived abilities in various domains and thus form specific self-concept configurations, which in turn might influence course selection. However, these studies incorporated only math and verbal (and other nonscience) self-concepts (Marsh et al., 2009; Umarji et al., 2018), only science self-concepts (Guo et al., 2017), or only a small selection of science and/or math self-concepts (Nagy et al., 2006). Drawing on the dimensional comparison theory (DCT; Möller and Marsh, 2013), we simultaneously considered self-concepts in various science and nonscience domains and investigated the patterns of self-concept combinations. To the best of our knowledge, we are the first to combine a person-centered approach with a multigroup approach to investigate such a large array of self-concepts relevant for science course selection in order to detect gender differences in these self-concept profiles. Furthermore, we investigated the relation of these profiles to science course selection and career aspirations. Finally, we take a long-term approach by not investigating males and females self-concepts at the time of academic choices toward science but by investigating the impact of early self-concept on later academic choices.

### Dimensional Comparisons and Structure of Self-Concept

Students’ self-concept is defined as being multifaceted as well as hierarchical and as the self-perception of an individual’s ability in a specific domain (Marsh, 2007). According to the DCT (Möller and Marsh, 2013), different dimensional comparisons between domains shape self-concept. Students compare their achievement in one domain with their achievement in another domain. This comparison either leads to self-concepts that are quite distinct from each other (high self-concept in one and low self-concept in the other domain; contrast effect) or to rather similar self-concepts (high self-concept in both domains; assimilation effect). Which effect occurs depends on the perceived subject similarity (Möller and Marsh, 2013; Möller et al., 2015). Rather dissimilar subjects are clearly distinct from each other, for example, subjects containing high levels of math content versus subjects containing high levels of verbal content. This phenomenon is also described as the math-verbal continuum (see Marsh et al., 1988). Research on comparison processes in self-concept formation has confirmed that math and verbal domains, such as German or a foreign language, constitute dissimilar subjects (Haag and Götz, 2012; Helm et al., 2016). Regarding the science and math domains, we assume that particular domains that are perceived as being more similar or less similar differently effect dimensional processes. In this regard, assimilation effects exist for math and physics while reading and physics operate as rather contrasting domains (Jansen et al., 2015). Looking at similarities between science subjects, one can assume that biology is more distinct from math than physics. Möller and Köller (2004) set up a model in which self-concept in physics and chemistry is assigned to an overarching mathematical self-concept factor, and biology self-concept is the only science domain that is assigned to an overarching verbal self-concept factor. Particularly biology and physics self-concepts serve as contrasting domains whereas physics and chemistry appear more similar to each other, thus resulting in assimilating dimensional comparisons (Guo et al., 2017).

Self-concept is reciprocally related to academic achievement; thus, individuals’ perception of their abilities is based on prior achievement and vice versa (e.g., Marsh and Craven, 2006). Therefore, achievement serves as an evaluator that shapes self-concept in each domain. School grades or scores of standardized achievement tests usually serve as indicators of achievement. School grades not only reflect a student’s academic achievement but also his or her relative position within the classroom setting. Grades are also a direct form of feedback for students regarding their achievement in a specific subject. In contrast, achievement scores in a standardized test are a criterion-referenced measure that is unbiased by group effects and not directly reported back to the student.

Most of the studies mentioned above applied a variable-centered approach (e.g., Nagy et al., 2006; Guo et al., 2017). Because within-person hierarchies of self-concept in various domains influence dimensional comparisons, person-centered approaches are also required. With person-centered approaches, individuals (in our case, students) with similar patterns regarding specific indicators are clustered together and students with disjunctive patterns are placed in different clusters (Lubke and Muthén, 2005). Hence, each student is categorized into a specific group according to his or her specific self-concept pattern. Thus, the approach attempts to carve out homogeneous groups within a heterogeneous population. Compared to the dominant variable-centered approaches, applying a person-centered approach can provide greater insights into students’ self-concept combinations. Most of the person-centered studies on motivational patterns used a set of motivational variables rather than self-concept alone (Lazarides et al., 2016; Linnenbrink-Garcia et al., 2018). To date, only a few studies have applied a person-centered approach to investigate self-concept patterns in students using various types of domain-specific self-concept (Marsh et al., 2009; Umarji et al., 2018). Marsh et al. (2009) applied a latent profile analysis (LPA) to a diverse set of academic self-concepts: math, verbal, problem solving, intellectual, artistic, political, technical, and computer self-concept, assessed in the final year of upper secondary school. They were able to distinguish between profiles that showed a combination of quantitative (high, low, and average overall self-concept) and qualitative differences among the single domains (e.g., high math self-concept and low verbal self-concept and vice versa). The authors identified four profiles, which reflected different levels of overall self-concept, that is, one low self-concept pattern, one medium self-concept pattern, and two high self-concept patterns. These profiles also showed opposing math and verbal self-concepts, indicating the verbal-math continuum based on the DCT (Möller and Marsh, 2013). Only one group had an average level of all self-concept indicators with rather similar math and verbal self-concept means. Similarly, a study by Umarji et al. (2018) incorporated the two contrasting domains of math and English (mother tongue) self-concept. The authors identified five groups of students in Grade 7 with three qualitative level differences among the two domains (e.g., low math—medium English, medium math—high English, or high math—medium English) and two groups of students that differed in terms of quantitative overall levels (low in both domains and high in both domains). Although the self-concept indicator as well as the number of profiles varied in both studies, there is evidence of at least two profiles representing contrasting domains (high math-related and low verbal-related vs. high verbal-related and low math-related). Moreover, both studies also identified groups that differed only in their quantitative level but did not show differences between math and verbal self-concept. The described studies on self-concept suggest that, within the framework of person-centered approaches, one can expect to find at least two profiles showing shape differences and two profiles showing level differences (overall high and overall low self-concept).

### Domain-Specific Self-Concept and Academic Choices Toward Science

Motivational variables such as self-concept are important predictors of course selection (Marsh and Yeung, 1997) and aspirations toward a career in science (DeWitt et al., 2013). Several studies have revealed that domain-specific self-concept predicts academic choices over and above prior academic achievement (e.g., Marsh and Yeung, 1997; Köller et al., 2000; Watt, 2006; Nagy et al., 2008; Wang and Degol, 2013). Students with a high self-concept in a STEM domain are more likely to remain on a STEM path even after it is no longer compulsory (Halpern et al., 2007). Students with a high math-specific self-concept are more likely to select math courses in high school (Nagy et al., 2006; Watt, 2006). The effects in math ranged between *OR* = 2.15 to *OR* = 2.95 after controlling for several cognitive variables in logistic regressions (Köller et al., 2000; Nagy et al., 2008). Comparable analyses were performed to predict the choice of STEM occupations and they showed that math-specific self-concept in the 12th grade was also one of the stronger predictors of this choice (*OR* = 1.46). Another study revealed that self-concept in science predicts aspirations for a science career (Nagengast and Marsh, 2012).

The presented studies incorporated only same-domain self-concept in order to predict academic choices in the respective domain, we not only considered self-concept and achievement in the chosen domain but also incorporated several domain-specific science and nonscience self-concepts to account for various comparison processes. Research on the DCT model has already proved the applicability of intraindividual dimensional comparisons in explaining academic choices while using variable-centered approaches (Nagy et al., 2006; Parker et al., 2012; Guo et al., 2017) as well as person-centered approaches (Wang et al., 2013; Umarji et al., 2018). Regarding the dual role of math and language as contrasting domains, results have revealed that students with a higher math self-concept relative to verbal self-concept are more likely to choose a math intensive major (Nagy et al., 2008; Parker et al., 2012; Guo et al., 2015; Umarji et al., 2018). This is also the case in dimensional comparisons between math and biology self-concepts and course selection in the respective subject. Students with a high self-concept in math were more likely to choose an advanced math course and less likely to opt for an advanced biology course (Nagy et al., 2006). Regarding science-specific comparisons, Guo et al. (2017) showed that self-concept in physics negatively predicted coursework aspirations in biology and vice versa. Taken together, science course selection is based not only on self-concept and achievement in a particular science domain. Rather, students seem to compare their achievement and self-concept with other domains such as reading as a rather dissimilar domain or math as a similar domain and thus evaluate their individual relative strengths. However, up until now, studies have not included a wide spectrum of science and nonscience self-concepts. Thus, a person-centered approach incorporating these various self-concepts could be especially fruitful to investigate whether students’ science course selection and career aspirations are associated with students’ intraindividual patterns of self-concept in various domains.

### Gender Differences in Self-Concept and Educational Choices

Although gender differences in certain abilities such as math and reading seem to have become increasingly negligible (Hyde, 2005; Else-Quest et al., 2010; Lindberg et al., 2010), gender differences in self-concept are still visible. A number of studies have revealed that, from early school years onward, the self-concepts of females and males differ depending on the academic domain (Herbert and Stipek, 2005; Spinath et al., 2014). Females, for instance, often have a lower positive self-concept in science and math than males do (Marsh and Yeung, 1998; Köller et al., 2000; DeWitt et al., 2013), whereas males show relatively lower self-concepts in verbal abilities (Schilling et al., 2006). This gender gap in the perception of one’s own abilities even exists after prior achievement has been controlled for (Wilkins, 2004; Sikora and Pokropek, 2012; Jansen et al., 2014). Thus, females seem to underestimate their abilities in math, which in turn might lower their math self-concept.

Taking a more differentiated look at science, gender differences vary across scientific fields (Britner, 2008). In physics, males tend to have a higher self-concept than females do (Schilling et al., 2006; Jansen et al., 2014). The results on self-concept in biology are mixed. While Schilling et al. (2006) found a higher self-concept in biology for males, other studies reported a higher self-concept in biology for females (Nagy et al., 2006; Jansen et al., 2014). Because gender differences do occur in terms of self-concept, different mechanisms behind males’ and females’ academic choices for and against STEM subjects could be derived from these self-concept differences. These different perceptions of one’s own abilities in a scientific field could lead to males choosing math- and science-related courses more often (Smyth and Hannan, 2006). Differences in self-concept are a key predictor in explaining gender differences in educational choices (Nagy et al., 2006; Watt, 2006). For instance, females with high abilities and a high self-concept in biology are more likely to choose advanced biology courses (Nagy et al., 2006). Whereas, for males, the same effect of math achievement and self-concept on math course selection is more pronounced than for females (Guo et al., 2015).

Taken together, self-concept not only differs across domains but also across gender. This effect has repeatedly been found in variable-centered studies. So far, studies have not tested for gender-specific profiles by combining a multigroup (gender) and a person-centered approach (homogeneous groups within one gender category). In such a framework, gender differences can mainly occur in two different ways. First, males and females can have different profile patterns, which lead to different choices regarding science. Second, both genders can show the same profile patterns, but the distribution across the profiles may differ across gender. Some studies have revealed that males were more likely to fall into a profile with high math self-concept, whereas females were more likely to be in a high verbal self-concept profile (Marsh et al., 2009; Umarji et al., 2018). However, the studies did not systematically test for gender invariance in profiles and did not apply these methods to investigate gendered paths to STEM, for example, academic choices toward science. The nature of profiles can differ across gender in terms of number of profiles, structure of profiles, as well as the relationship between profiles and predictors, or outcomes. Thus, taking a multigroup perspective within a person-centered approach into account not only helps to systematically assess gender differences in the nature of profiles but also to fully disentangle gendered pathways into science choices and career aspirations. We took this innovative approach and investigated several science and nonscience self-concepts that may lead to staying in or dropping out of science courses. In addition, we took a new approach by focusing not on self-concept at the time of the academic choice but on the long-term effects of self-concepts for these academic choices.

### The German Secondary School System

Despite each federal state in Germany having its own education system, the systems also share common features. First, science education takes place in separate courses for biology, chemistry, and physics, and typically starts in Grade 7. Prior to Grade 5, elementary schools provide a combined course that includes social science, history, geography, science, and technology. In the federal state of Hamburg, where our study took place, in Grades 5 and 6, academic secondary schools offer an integrated science/technology course. In Grade 7, the structure changes to separate courses in biology, chemistry, and physics. Second, when entering upper secondary education (10th grade) students have to choose advanced courses that they would like to focus on and basic courses that they wish or need to continue. These choices take place in track systems or course systems, depending on the federal state. Hamburg has a track system. At the end of Grade 10, students have to choose between five tracks, namely, science/technology, language, social sciences, arts, and sports.

### The Present Study

The aim of the present study was to identify males’ and females’ self-concept profiles using LPA. Further, we incorporated several relevant predictors to account for the influence of achievement and outcome variables to fully discern the mechanisms behind gendered differences in self-concept profiles and in academic choices toward science. Unlike previous studies, we incorporated a wider range of science and nonscience self-concepts, namely, math, verbal, biology, and physics; we also focused on the impact of early self-concept on later academic choices.

First, we examined students’ self-concept profiles across the domains of math, biology, physics, German, and English, separately for each gender. Applying a person-centered approach, we expected to find different profiles that show qualitative and quantitative self-concept differences. We expected to find profiles that demonstrate the verbal-math continuum based on the DCT (Möller and Marsh, 2013). Therefore, we assumed that at least four profiles would emerge. We expected to find two qualitatively different profiles; one profile showing high self-concept values in the math-related domains of math and physics, and low self-concept values in the verbal-related domains of English and reading; and a second profile showing low self-concept values in the math-related domains and high self-concept values in the verbal-related domains. Because results concerning the placement of biology on the math-verbal continuum are mixed, we did not formulate a specific hypothesis regarding the assignment of biology. Studies investigating patterns of motivational variables have demonstrated that profiles also differ in terms of quantity (high and low overall self-concept, in some instances, a medium overall self-concept), in which the values of math-related and verbal-related self-concepts are rather similar (Marsh et al., 2009; Umarji et al., 2018). Hence, we also expected to find at least two profiles, one with overall low self-concept values and one with overall high self-concept values.

Second, applying the novel approach for testing gender invariance in LPA, we systematically assessed whether the profiles were similar across gender (Morin, 2016). We did not specify hypotheses regarding differences between males and females due to the lack of studies using the person-centered approach to investigate the profiles of domain-specific self-concepts across gender in a multigroup framework. Thus, we conducted an explorative test regarding differences in the number of profiles and the qualitative differences of profiles across gender. However, research regarding gender differences in self-concept has shown that males have higher self-concept values in math-related domains and females have higher self-concept values in verbal-related domains. Hence, we expected that proportionally more females would be in profiles characterized by a high verbal self-concept whereas males would more frequently belong to profiles characterized by a high math self-concept.

Third, achievement in the respective domain influences the formation of students’ self-concept and students rely on achievement in different domains to shape their self-concept. Therefore, we hypothesized that high achievement in math domains and low achievement in verbal domains would be positively associated with high math-related self-concept (HMRSC) profiles whereas a low math and high verbal achievement constellation would be positively associated with profiles characterized by high verbal-related self-concept (HVRSC) profiles. Due to our novel approach, which combined the person-centered and multigroup perspective, we were not able to formulate hypotheses regarding gender differences within the relations between achievement and self-concept.

Finally, we expected that group membership would predict science course selection and science aspirations. More precisely, we hypothesized that students in high math- and science-related self-concept profiles would be more likely to choose a science course and hold higher aspirations toward science occupations than students in high verbal self-concept profiles. Because several studies have revealed that males are more likely to choose a science course than females in upper secondary school, we hypothesized that, in profiles with quite identical math and verbal self-concept values, females would be more likely to choose science courses.

## Materials and Methods

### Procedure and Sample

We performed secondary analyses on a subsample of the longitudinal “Competencies and Attitudes of Students” study [KESS] (Bos and Gröhlich, 2010) conducted in the German federal state of Hamburg. The sample of the KESS study comprised various school types. We investigated the subsample of 1,658 students attending 61 academic schools (*Gymnasium*). Only this school type leads to upper secondary education and comprises 3 years more than the other school types. We used data from the measurement points in Grade 8 and Grade 12, measured in 2007 and 2011, respectively. Domain-specific self-concept, science aspirations, and ability in various domains (achievement test scores and grades) were measured at the end of the eighth grade. In the 12th grade, students stated which track they selected in Grade 10.

### Measures

#### Domain-Specific Self-Concept

Students’ domain-specific self-reported perception of their abilities was assessed with separate scales for each domain. Self-concept in biology, physics, and math was each measured using three scales adopted from the Academic Self-Description Questionnaire II scale (ASDQ II; Marsh, 1990). Each scale comprised three items, for example, “I have always been good in [biology/physics/math.]” (biology: α = 0.87, physics: α = 0.90, and math: α = 0.93). Self-concept in reading (German) was assessed using seven items adopted from the Progress in International Reading Literacy Study (PIRLS; Kelly, 2003), for example, “I have difficulties understanding texts.” (α = 0.75). Self-concept in English was assessed using five items adopted from Jerusalem (1984). A typical item was: “Nobody can be good in every subject. I am just not talented in English” (α = 0.91). All items were rated on four-point Likert-type scales.

#### Science Track Selection

Students indicated their course choices for Grades 11 and 12. We used the choice of the first course as an indicator of the choice of a science or nonscience track (values 1 and 0). The subject choices biology, chemistry, and physics indicated science track selection. All remaining first course choices were an indicator of nonscience track selection.

#### Science Aspirations

On a four-point Likert-type scale, students answered an item asking whether they were interested in choosing an occupation in a scientific field after completing school. The item was measured in Grade 8.

#### Standardized Achievement Test

Students’ achievement in science, math, and reading was measured using standardized achievement tests covering the curriculum in the respective domain. Applying an anchor-item design and IRT scaling across the three measurement points ensured the comparability of the achievement test scores (Davier and Davier, 2007). A common longitudinal metric was built (*M* = 100, *SD* = 30). All reliabilities reported in the manuals of the respective studies were satisfactory (Bos et al., 2010).

#### School Grades and Gender

Students indicated their grade in math, German, English, biology, and physics from the latest school report card. School grades in lower secondary level range between one and six, with one being the highest score. We recoded the grades for the sake of clarity, thus, higher scores reflect higher achievement. Finally, students reported their gender (0 = male, 1 = female).

### Statistical Analyses

All analyses were conducted in M*plus* 7.4 (Muthén and Muthén, 1998–2015) using maximum likelihood with robust standard errors (MLR). Our initial analyses included correlation analyses and tests for mean differences between males and females. The data set contained data missing at random in the indicator variables for the LPA as well as in the covariates. Therefore, we handled the missing data with the imputation function in M*plus* and produced 50 imputed data sets (Graham et al., 2007; Enders, 2010; Hickendorff et al., 2018). We integrated these 50 data sets with the function TYPE = IMPUTATION. We also took the hierarchical data structure into account by adjusting standard errors using the TYPE = COMPLEX option in M*plus*, specifying schools as clusters. LPA was applied using *z*-standardized domain-specific self-concept indicator variables.

In the first step, we examined the number of profiles for males and females independently in consecutive LPAs. In each model, indicator means were freely estimated while the variance of the indicators was held equal across profiles^1^. Models were estimated based on 5,000 random sets of start values and 100 iterations per start. The decision for an optimal profile solution was based on a variety of statistical fit indexes. Thus, we evaluated the Akaïke information criterion (AIC), the consistent AIC (cAIC), the Bayesian information criterion (BIC), and the sample-adjusted BIC (SABIC). Simulation studies recommend the use of the cAIC, BIC, and SABIC (e.g., Nylund et al., 2007; Tofighi and Enders, 2008). Lower values indicate a better fit. As the fit indices in large sample sizes frequently improve with the number of profiles, we also inspected elbow plots to detect the point at which the decrease in the fits became negligible (Morin et al., 2016a). We also considered the theoretical meaning of the profile solutions (Muthén, 2003; Marsh et al., 2009) as profiles should be meaningful and interpretable as well. Hence, we inspected the self-concept composition of additional profiles. We preferred models with fewer profiles when an additional profile was quite similar to the already existing profiles. Finally, the entropy and the average posterior probabilities, varying from 0 to 1, gives information about how well students can be classified to their most likely profile. The higher the value, the smaller the classification error. Nagin (2005) suggests an average posterior probability value of at least 0.70 as the cutoff for an acceptable classification error. In the following steps, we used a multigroup approach using the KNOWNCLASS function to investigate whether gender differences occurred in (a) the nature of the profiles, (b) the prediction, and (c) the outcome (Morin, 2016; Morin et al., 2016b).

In the second step, we examined the degree of similarity across gender in a sequence of models after identifying the final number of profiles for males and females. First, a baseline model with freely estimated profile-specific indicator variables across gender was estimated (configural model). Then, the models were successively constrained with regard to profile indicators across gender (i.e., means and variance). Thus, we tested whether the means within the profiles were similar across gender (structural similarity) and we tested whether the variance within profiles were similar across gender (dispersion similarity). Finally, we examined whether the relative profile sizes were equal across gender (distributional similarity). Models with equality constraints across gender were compared to a less restrictive model using cAIC, BIC, and SABIC. Lower values on at least two information criteria indicated a favorable model (Morin, 2016).

In the third step, we added predictors to the final model. A multinomial logistic regression was used to predict profile membership. We further tested for differences across gender by comparing a model constraining logistic regression coefficients to be equal between males and females (predictive similarity) with a model where coefficients were freely estimated between males and females. We then compared the model fit. In a last step, we added the outcome variables, science course selection, and science aspirations to the final model. Again, we first estimated a model with freely estimated within-profile outcomes across gender and compared the model to a model with equality constraints of the outcome variables across gender (explanatory similarity). To ensure that including covariates and outcomes did not change the profile solution, all models were estimated applying the manual three-step method (Asparouhov and Muthén, 2014; Morin and Litalien, 2017). Moreover, if the model with freely estimated coefficients between males and females was superior, we further checked pairwise differences of coefficients across profiles and gender using the model constraint function.

## Results

Before proceeding to the hypothesis testing, we report the descriptive findings of self-concept and the achievement means of males and females (see Table 1). Correlations are reported in the Appendix (see Appendix Tables A1, A2). The *t*-test and effect sizes show that gender differences in favor of males occurred for self-concept in physics and math. The effect sizes for both are quite substantial.

**Table 1:** Means of males’ and females’ self-concepts as well as achievement in different domains.

	Males (*n* = 714)	Females (*n* = 944)	Cohen’s *d*	Mean Test between- gender difference
**Self-concept**	**Mean (*SD*)**	**Mean (*SD*)**		***T*-Test**

Biology	3.32 (0.74)	3.31 (0.65)	0.01	0.29
Physics	3.19 (0.79)	2.72 (0.80)	0.59	11.97***
German	3.30 (0.50)	3.31 (0.49)	0.001	0.29
Math	3.33 (0.75)	2.85 (0.84)	0.59	11.91***
English	3.23 (0.75)	3.35 (0.68)	0.17	3.49***
**Achievement**				
**Test scores**				
Science	155.83 (31.23)	147.39 (28.24)	0.29	5.76***
Reading	150.94 (18.94)	159.79 (16.78)	0.50	10.05***
Math	165.03 (23.26)	159.22 (21.43)	0.26	5.27***
English	144.86 (20.72)	151.11 (18.26)	0.30	6.51***
**Grades**				
Biology	4.21 (0.84)	4.34 (0.84)	0.16	3.31***
Physics	4.23 (0.90)	4.26 (0.90)	0.03	0.69
German	4.05 (0.82)	4.44 (0.76)	0.49	9.85***
Math	4.13 (0.99)	4.16 (0.99)	0.04	0.77
English	3.94 (0.92)	4.31 (0.89)	0.41	8.26***

*^∗∗∗^*p* < 0.001; ^∗∗^*p* < 0.01; ^∗^*p* < 0.05; all achievement variables were measured in Grade 8.*

Females showed higher English self-concept than males but the effect size shows that the difference had only little practical relevance. Many gender differences occurred regarding achievement. Males had significantly higher scores in standardized achievement tests in math and science whereas females showed higher scores in reading and English. However, the effect sizes indicated a medium effect for reading whereas the remaining domains showed rather small effect sizes. With regard to school grades, math and physics did not significantly differ across gender. Females received significantly better grades in the verbal domains.

### Males’ and Females’ Self-Concept Profiles

We ran consecutive LPAs from one to six profiles. Table 2 displays the fit indices, average probabilities of profile membership, and classification accuracy (entropy) for the subsamples males and females. The normality and local independence assumptions of LPA were met.

**Table 2:** Results from the latent profiles analyses.

Model	LL	# of par.	AIC	cAIC	BIC	SABIC	ϕ Profile prob.	entropy
Females								
1 profile	-6697.39	10	13414.78	13473.28	13463.28	13431.52	n.a.	n.a.
2 profile	-6520.61	16	13073.21	13166.81	13150.81	13100.00	>0.88	0.66
3 profile	-6441.81	22	12927.62	13056.32	13034.32	12964.45	>0.81	0.69
4 profile	-6403.64	28	12863.29	13027.09	12999.09	12910.17	>0.76	0.70
5 profile	-6360.91	34	12789.82	12988.72	12954.72	12846.74	>0.79	0.76
6 profile	-6352.72	40	12785.44	13019.44	12979.44	12852.40	>0.80	0.79
Males								
1 profile	-5065.61	10	10151.22	10206.93	10196.93	10165.18	n.a.	n.a.
2 profile	-4911.66	16	9855.32	9944.46	9928.46	9877.65	>0.90	0.69
3 profile	-4863.50	22	9771.00	9893.56	9871.56	9801.70	>0.81	0.69
4 profile	-4837.89	28	9731.78	9887.77	9859.77	9770.86	>0.76	0.72
5 profile	-4817.99	34	9703.98	9893.39	9859.39	9751.43	>0.69	0.68
6 profile	-4802.44	40	9684.89	9944.46	9867.72	9740.71	>0.71	0.70

*LL, log likelihood; # of par., number of free parameters; AIC, Akaïke information criteria; CAIC, constant AIC; BIC: Bayesian information criterion; SABIC, sample-size adjusted BIC; n.a., not applicable.*

In both subsamples, the fit indices continuously decreased as the number of profiles increased up to the fifth profile. The cAIC, BIC, and SABIC increased, thus, the sixth profile was statistically unsatisfactory. However, we also inspected a graphical representation of the fit indices. The indices attenuated at the fourth profiles (see Appendix Figures A1, A2). Adding a fifth profile did not result in a further meaningful profile. In fact, this fifth profile resembled an existing profile (see Appendix Figures A3, A4). In both samples, the 4-profile solution showed sufficient classification accuracy (entropy). The average probabilities of profile membership in the profiles were high and ranged between 0.76 and 0.85 in the females’ subsample and between 0.76 and 0.87 in the males’ subsample. Thus, the models can distinguish quite well between the profiles. Taking these fit values into account, as well as the theoretical foundations of the profiles, and the inspection of the emerging profile in the 5-profile solution, the 4-profile solution was retained for subsequent analysis in both samples.

### Analyses of Profile Similarities Between Males and Females

The LPA of each subsample revealed the same number of profiles for males and females. Thus, in a second step, we investigated the profile similarity between males and females using a multigroup approach. Table 3 shows the fit indices for the models. First, a 4-profile model with freely estimated means across subsamples was estimated (configural model). The configural model was compared with a more restrictive model in which the means were held equal across gender (structural model).

**Table 3:** Test of profile similarity, predictive similarity, and explanatory similarity.

	LL	# of par.	AIC	cAIC	BIC	SABIC
Profile similarity						
Configural: All means free	-12264.12	57	24642.23	25007.79	24950.80	24769.71
Structural: All means equal	-12318.68	37	24711.37	24948.66	24911.66	24794.20
Dispersion: All equal	-12328.13	32	24720.26	24925.49	24893.49	24791.83
Distributional	-12373.87	29	24805.74	24991.73	24962.73	24870.60
Predictive similarity: test score						
Freely estimated	-2897.35	31	5856.70	6055.52	6024.52	5926.04
Equality across gender	-2904.96	19	5847.91	5969.77	5950.77	5890.41
Predictive similarity: grades						
Freely estimated	-2706.43	37	5486.85	5724.15	5687.14	5569.60
Equality across gender	-2718.08	22	5480.16	5621.26	5599.26	5529.37
Explanatory similarity						
Freely estimated	-5989.74	25	12029.48	12189.810	12164.81	12085.39
Equality across gender	-6055.97	17	12145.95	12254.975	12237.98	12183.97

*LL, log likelihood; # of par., number of free parameters; AIC, Akaïke Information Criteria; CAIC, Constant AIC; BIC, Bayesian Information Criterion; SABIC, Sample-Size Adjusted BIC; n.a., not applicable.*

The structural model resulted in lower cAIC and BIC values, indicating that the four profiles showed similar within-profile means across gender. Next, a model with means and within-profile variance held equal across gender was estimated (dispersion model). This model resulted in lower fit indices than the prior model and thus further supports the structural profile similarities across gender. Finally, the distributional model was estimated by constraining the profile sizes of the profiles to be equal across gender. All fit indices were higher than in the dispersion model, indicating that the relative size of the latent profiles was not similar across gender, which means that the profile membership differed as a function of gender.

**FIGURE 1:**
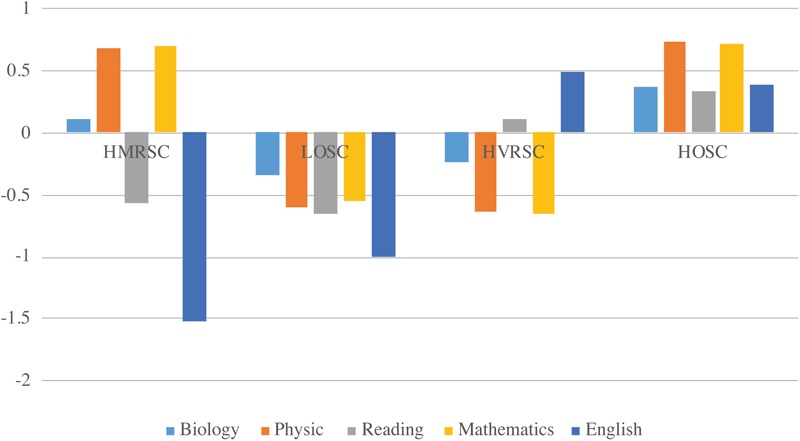
Final profile solution. HMRSC, high math-related self-concept; LOSC, low overall self-concept; HVRSC, high verbal-related self-concept; HOSC, high overall self-concept profile.

The final multigroup 4-profile model, with equal within-profile means and within-profile variability across gender but freely estimated profile probabilities was retained for further analysis. Figure 1 exhibits the profiles and Table 4 the gender distribution across the four profiles.

**Table 4:** Gender distribution across profiles.

	High math-related self-concept	Overall low self-concept	High verbal-related self-concept	Overall high self-concept
Males	19%	11%	28%	43%
Females	7%	18%	48%	28%

The first profile reflects high math-related self-concepts (math and physics) and low verbal-related self-concepts (German and English). We labeled this profile *high mathrelated self-concept* (HMRSC). As we hypothesized, this group included 19% of the males but only 7% of the females. Even though we had refrained from formulating hypotheses regarding biology self-concept, we found that this domain-specific self-concept did not show directionality on the math-verbal continuum. The second profile (11% males and 18% females) comprised low levels of self-concept across all domains with rather identical physics, math, and reading self-concept. Biology self-concept, though still low, was slightly higher than the remaining self-concepts whereby English self-concept was slightly lower than all other self-concepts. We labeled this profile *low overall self-concept* (LOSC). The third profile showed differences between math-related and verbal-related self-concepts and was somewhat the opposite of the HMRSC profile. Students in this profile had high verbal self-concepts (German and English) and low math-related self-concepts (physics and math). Self-concept in biology did not show directionality toward math-related self-concept on the verbal-math continuum. Thus, we labeled the profile *high verbal-related self-concept* HVRSC. As we expected, this profile was prevalent for almost half of the females (48%) but only for 28% of the males. Finally, the last profile constituted most of the students and contained 43% of the males’ subsample and 28% of the females’ subsample. The profile was characterized by high math and physics self-concept, which was slightly larger than reading, biology, and English self-concept. This configuration corresponds to a *high overall self-concept* profile (HOSC). As we had expected, these four profiles represent level (LOSC and HOSC) and shape differences (HMRSC and HVRSC). Therefore, again as we expected, we found profiles that differed both quantitatively and qualitatively.

We also conducted a chi-square analysis and adjusted standardized residuals to investigate gender differences in profile membership (see Appendix Table A3). As hypothesized, gender differences occurred in all profiles following a stereotypical pattern. Females were overrepresented in the HVRSC and the LOSC profile whereas males were overrepresented in the HMRSC profile and the HOSC.

### Predictive Similarity

We added covariates to the final multigroup model to investigate the prediction of profile membership via multinomial regression. A multigroup approach was used to investigate whether covariates differentially predicted profile membership for males and females. We analyzed two sets of covariates: achievement in a standardized test and school grades. We again compared two models, a model freely estimating the predictors across gender and a model constraining the paths to be equal across gender. The constrained model resulted in lower fit values, indicating predictive similarity across gender. Table 5 presents the results of the multinomial logistics regression. We report odds ratios (OR). ORs reflect the change in the likelihood of being in a profile versus a comparison profile for each unit of increase in the predictor. An OR of 1 indicates that the likelihood to be placed in a profile versus a comparison profile is equal, and an OR above 1 indicates a higher likelihood to be placed in the comparison profile.

**Table 5:** Relations of achievement measures to self-concept profile membership (Multinomial logistic regression, separate analyses for test scores and school grades).

	HMRSC vs. LOSC	HMRSC vs. HVRSC	HMRSC vs. HOSC	LOSC vs. HOSC	LOSC vs. HVRSC	HVRSC vs. HOSC
	**OR (SE_b_)**	**OR (SE_b_)**	**OR (SE_b_)**	**OR (SE)**	**OR (SE_b_)**	**OR (SE_b_)**
Achievement test scores						
Math	1.07 (0.02)^∗∗∗^	1.07 (0.01)^∗∗∗^	1.01 (0.01)	0.95 (0.01)^∗∗∗^	1.00 (0.01)	0.95 (0.01)^∗∗∗^
Reading	0.99 (0.01)	0.99 (0.01)	0.99 (0.01)	1.00 (0.01)	1.00 (0.01)	1.00 (0.01)
Science	1.03 (0.01)^∗∗^	1.03 (0.01)^∗∗∗^	1.00 (0.01)	0.98 (0.01)^∗∗∗^	1.03 (0.01)^∗^	0.97 (0.01)^∗∗∗^
English	0.95 (0.02)^∗∗^	0.89 (0.01)^∗∗∗^	0.93 (0.01)^∗∗∗^	0.96 (0.01)^∗∗∗^	0.97 (0.01)^∗∗^	1.04 (0.01)^∗∗∗^
School grades						
Math	10.97 (0.41)^∗∗∗^	10.79 (0.24)^∗∗^	1.64 (0.24)^∗^	0.26 (0.25)^∗∗∗^	1.68 (0.23)^∗^	0.15 (0.20)^∗∗∗^
German	0.56 (0.30)^∗∗∗^	0.51 (0.22)^∗^	1.21 (0.21)	2.31 (0.23)^∗∗∗^	0.99 (0.21)	2.35 (0.18)^∗∗∗^
Biology	1.67 (0.31)	1.32 (0.22)	0.90 (0.23)	0.58 (0.22)^∗^	0.85 (0.20)	0.69 (0.19)^∗^
Physics	4.17 (0.33)^∗∗∗^	5.28 (0.24)^∗∗^	1.16 (0.20)	0.30 (0.22)^∗∗∗^	1.36 (0.18)	0.22 (0.18)^∗∗∗^
English	0.23 (0.47)^∗∗∗^	0.06 (0.30)^∗^	0.11 (0.29)^∗∗∗^	0.28 (0.28)^∗∗∗^	0.15 (0.22)^∗^	1.94 (0.24)^∗∗^

*^∗∗∗^*p* < 0.001; ^∗∗^*p* < 0.01; ^∗^*p* < 0.05; all achievement variables were measured in Grade 8. OR, odds ratio; SE_*b*_, standard error of beta; HMRSC, high math-related self-concept; LOSC, low overall self-concept; HVRSC, high verbal-related self-concept; HOSC, high overall self-concept profile.*

Students with high achievement test scores in math and in science were more likely to belong to the HMRSC profile rather than to the LOSC profile or the HVRSC profile. Students with lower achievement test scores in math and science were more likely to belong to the HVRSC profile than to the HOSC profile. Students with high English achievement test scores were more likely to belong to the HVRSC profile than to the HOSC profile. Students with low English achievement test scores were more likely to be in the HMRSC profile than in any of the three others or were more likely to be in the LOSC profile than the HVRSC profile.

The effects of school grades on group membership revealed similar results. Again, students with higher grades in math and in physics were more likely to be members of the HMRSC profile in comparison to the LOSC profile and the HVRSC profile. Lower grades in math and in physics led to higher membership chances in the HVRSC profile and the LOSC profile compared to the HOSC profile. Students with better grades in German and in English were more likely to be a member of HVRSC profile than the HOSC profile. Students with lower grades in English were more likely to belong to the HMRSC in comparison to all other profiles. However, students with lower grades in English were also more likely to be in the LOSC profile than in the HOSC profile or the HVRSC profile. Regarding grades in biology, students with lower grades had a higher likelihood of membership in the HVRSC and in the LOSC profiles compared to the HOSC profile.

In a last step, we added outcome variables to the final model. Again, we compared a model with freely estimated mean-level outcomes with a model in which the mean-level outcomes were constrained to be equal across gender. We tested the models for each of the outcome variables. Table 3 shows that the fit indices were lower for the freely estimated model, thereby indicating that course selection varies as a function of gender.

Pairwise mean differences were tested between the profiles and across gender (Table 6). The likelihood of choosing a science course at the beginning of upper secondary school was highest in the HMRSC profile and in the HOSC profile as compared to the LOSC and HVRSC self-concept profile. This was true for both males and females. However, in the HMRSC profile and in the HOSC profile, the probability of choosing a science course was lower for females than for males. We found the same pattern for science aspirations, indicating that students in the HMRSC and HOSC profile were more likely to choose a science career in the future. However, with regard to gender differences, only the HOSC profile revealed significant differences, indicating that males’ aspirations for a science career were higher than females’.

**Table 6:** Associations between profile membership course selection and career aspirations in science.

Outcome	HMRSC (1)	LOSC (2)	HVRSC (3)	HOSC (4)
	**OR**	**OR**	**OR**	**OR**
Science course male	1.81 (0.11)^2,3^	1.20 (0.08)^1,4^	1.17 (0.03)^1,4^	1.52 (0.06)^2,3^
Science course female	1.32 (0.06)^2,3,a^	1.13 (0.03)^1,4^	1.13 (0.02)^1,4^	1.29 (0.03)^2,3,a^

	**Coef. (SE)**	**Coef. (SE)**	**Coef. (SE)**	**Coef. (SE)**

Science aspirations male	2.94 (0.11)^2,3^	2.03 (0.17)^1,4^	2.00 (0.06)^1,4^	2.88 (0.08)^2,3^
Science aspirations female	2.91 (0.11)^2,3,4^	2.13 (0.11)^1,4^	1.89 (0.06)^1,4^	2.613 (0.06)^2,3,1,a^

*^*a*^significant differences between gender, *p* < 0.05. Indices indicate the other profile that has a significantly different value compared to the respective profile, for example: 1.81^*2,3*^ of HMRSC profile (1) is significantly higher than 1.20 of LOSC profile (2) and higher than 1.17 of HVRSC profile (3). HMRSC, high math-related self-concept; LOSC, low overall self-concept; HVRSC, high verbal-related self-concept; HOSC, high overall self-concept profile.*

## Discussion

The focus of our study was to explore gendered differences within self-concept profiles and their relation to pursuing a scientific path. In order to gain deeper insights into choices made regarding science at upper secondary school, we examined the joint effects of prior domain-specific self-concept (i.e., math, reading, physics, biology, and English) on course selection in secondary education. By adopting a person-centered approach, we explored how dimensional comparisons between these science and nonscience school subjects lead to science course selection instead of only taking a single domain into account. In contrast to previous studies, we incorporated these self-concepts to focus on science course selection and science aspirations, to systematically check for gender invariances within the person-centered approach, and to evaluate whether gender-specific differences in choosing a science path occur. We first conducted an LPA to analyze self-concept profiles separately for males and females and we then adopted a multigroup LPA to further investigate gender differences in profiles, profile membership, predictors, and outcomes.

### Self-Concept Profiles of Males and Females

As expected, the LPA revealed four distinct profiles that differed qualitatively (in shape) and quantitatively (in level). Thus, already in Grade 8, students develop a differentiated pattern of science and nonscience self-concepts. Three profiles reflected the verbal-math continuum. The first profile is characterized by high self-concept in math and physics and low self-concept in the verbal domains (i.e., reading and English). The opposing profile reflected high verbal self-concept and low math self-concept. One profile reflected a high overall self-concept across all domains, but with slightly higher math-related self-concepts compared to verbal-related self-concepts. The remaining profile reflected overall low levels of self-concept across all domains. In all profiles, math and physics self-concepts had comparable means within each profile. Biology, a self-concept component we explored without formulating hypotheses, could not be related to any end of the verbal-math continuum. This self-concept showed different positioning in the profiles. It rather belonged to the math-related self-concepts in the HVRSC profile, to the high verbal-related self-concepts in the HOSC profile, whereas, in the HMRSC and the LOSC profile, no clear allocation to the continuum could be made. Therefore, our results did not support the assumption that biology self-concept belongs to either one of the ends of the verbal-math continuum. Regarding the placement of biology self-concept within the verbal-math continuum, we might have revealed a reason for the inconsistent results from previous variable-centered studies. Whether or not biology can be placed on the verbal end of the verbal-math continuum might depend on the students’ self-concept profile. The nature of our profiles was similar to profiles identified in earlier studies; however, the number of profiles differed as the number of domain-specific self-concepts used in former studies differed as well (Marsh et al., 2009; Umarji et al., 2018). Thus, our results confirm the importance of an individual’s self-evaluation of strengths and weaknesses, which has already been established in the context of the DCT (Möller and Marsh, 2013).

The shape of the profiles was similar across gender, indicating that both genders had the same structure of self-concept profiles. We did not find distinctive self-concept patterns for males and females. Even though we did not find gender distinctive patterns, as expected, we did find differences in the distribution across profiles as a function of gender. The HVRSC profile and the LOSC profile were more frequent among females than males. In contrast, males were overrepresented in the profiles with higher math-related self-concepts (i.e., the HOSC profile and the HMRSC profile). About 62% of the males either had a high overall self-concept or a HMRSC, while this was the case for less than 35% of the females. The distributional differences suggest that a specific group of males’ and females’ self-concept should be strengthened according to their profile as early as Grade 8. As this was only 1 year after the beginning of science teaching in Germany, our results show that self-concepts should be fostered from the very beginning of teaching these subjects. In order to keep females on the science path, specific interventions should foster the math and physics self-concepts of females in the HVRSC profile and in the LOSC profile.

### Predictors

Stereotypical gender differences emerged in the achievement indicators. Although recent studies underline that gender differences in achievement almost disappear between males and females, contrary to the self-concepts, where we found more gender differences for physics and science, we found substantial differences between achievement indicators mainly for the verbal domains (i.e., German and English). Interestingly, we did not find any gender differences for grades in math and physics whereas males showed higher test scores in math and science than females. Moreover, the dimensional comparisons of the students’ achievement seemed to influence membership in the self-concept profiles. Just as has been shown for math, English, and German, we again showed that students rank their achievement in comparison to other domains in order to get information about their relative abilities in a specific domain (Marsh, 1990; Möller and Marsh, 2013) and this comparison forms their self-concept pattern. Students with high math and low verbal abilities rather belong to the HMRSC profile than to the HVRSC profile and vice versa. Interestingly, students with low abilities in English fall into the HMRSC profile rather than into the HOSC. Therefore, English (as a foreign language) seems to be a more contrasting domain in finding one’s own self-concept profile than the more general German (mother tongue) comparison. Hence, the perception of one’s abilities in the foreign languages is a quite important (negative) predictor of females staying on the STEM path. Moreover, as expected, the grade in math is the most powerful predictor of membership in the HMRSC profile for both males and females. Even though males and females did not differ regarding their math grades, females were less likely to be in profiles with HMRSCs. One explanation might be the attributional gender bias: males’ success in math is attributed to ability whereas females’ success is attributed to effort (e.g., Räty et al., 2002). Perhaps, females might not use their actual math grade to examine their abilities. Thus, other mechanisms seem to influence gender-specific distribution across profiles. Exploring these mechanisms should be the focus of future studies.

### Outcome

Students may be more likely to persevere in a subject in which they feel more competent than in another subject (Jonsson, 1999; Uerz et al., 2004). For instance, Marsh and Yeung (1997) were able to show that domain-specific self-concept predicts the choice of that particular subject more than self-concept in other subjects. Moreover, students tend to choose a science course more frequently when their abilities in math are clearly higher than their abilities in reading (Uerz et al., 2004). Our results support these assumptions. As we expected, students belonging to profiles with math-related self-concepts more frequently chose a science course and had higher aspirations toward a science career than students in the LOSC and the HVRSC profile. Because both profiles with math-related self-concepts drives science course selection, dimensional comparisons might lead and might not lead to science course selection at the time. Not only students with higher self-concept in mathematics compared to the verbal domain choose science courses but also students with high self-concept in both domains. This effect was even stronger for males in two ways. First, males tended to be in the two profiles favoring science course selection and science aspirations more often. Second, compared to females, males in the HMRSC or the HOSC profile more frequently chose a science course than females. In particular, females who perceived themselves as being good in both math and verbal subjects were less likely to choose science course than males. Having high abilities or self-concept in several domains leads to a broader range of possibilities in educational choices. Students might choose academic courses and later careers according to stereotypical interests or fields to which their environment directs them. For example, stereotypes regarding science as a male domain (Cheryan et al., 2011) and gender-role behaviors in students’ environment (Eccles, 1993) might influence educational decisions. These mechanism for this specific group needs to be addressed in future studies in order to tailor future interventions to keep females on the scientific path. Even though our study did not lead to specific interventions, we were able to show that there cannot be one strategy to keep females on this path. Furthermore, we showed that – just as for males – the group of females dropping out of science careers is not a homogenous group, just as the group of females that pursues a scientific career is not homogenous. Understanding the mechanisms behind the specific groups is an important task for future interventions. Our conclusion that females within specific profiles should be supported in strengthening their math- and physics-related self-concepts should ideally also result in females’ increased interest in a scientific career as well as in the choice of science courses during upper secondary education. Our study showed that already in Grade 8 self-concept predicts career aspirations. Although we were able to reveal this mechanism for STEM paths, future studies could investigate whether these mechanisms also appear for other paths such as choosing humanities or languages for males and for females.

### Limitation and Future Directions

Some limitations, which future studies could attempt to overcome, are worth mentioning. First, our study focused on general science course selection. The subsamples of students who chose advanced physics and chemistry courses were too small because these two school subjects are not very popular in Germany. Therefore, analyses of these fine-grained levels were not feasible. Future studies might look at how self-concept patterns (e.g., covering the differential self-concepts of our study) differentially influence the uptake of physics, biology, and chemistry courses. It would be interesting to investigate the impact of self-concepts that include high math contents (such as physics, which we were able to place on the verbal-math continuum, in comparison to biology) on science course selection.

Some methodological limitations are to mention. Similar to previous studies, we assessed domain self-concept with separate domain-specific Likert scales (i.e., for biology, physics, math, English, and German). Additionally, we could not rely on paralleled items across all scales. This approach may have the drawback that it was not able to measure exactly the same construct in all of the domains. This is a methodological problem that should be addressed in the future, especially in the light of DCT studies. Further, the entropy values in the female and male subsamples were about 0.70 suggesting that precision of classification might not be highly accurate. However, the average posterior probabilities ranged between 0.76 and 0.88. The cross probabilities values, which ranged between 0.01 and 0.16, indicated that students had also a 1–16% chance of belonging to another profile. Thus, the chosen model can distinguish quite well between the profiles.

Future studies could also focus on the predictors of self-concept patterns. We showed that individuals’ achievement is a source of self-concept formation but peers, teachers, and family also play an important role in perceptions of self-concept. Previous studies have already demonstrated that school and family influence career choices (Mau and Bikos, 2000; Fouad and Santana, 2017). The shaping of self-concept can provide valuable information about why females are less likely to choose STEM. Moreover, future studies could incorporate a wide array of motivational variables that might explain gender differences in course selection, such as task values, interest, and self-efficacy (Guo et al., 2015; Korhonen et al., 2016; Lin et al., 2018; Linnenbrink-Garcia et al., 2018) and these individual predictors could be combined with school, family, and peer predictors. Incorporating these additional variables and utilizing the DCT as well as the person-centered and the multigroup approach could reveal whether the same mechanisms apply to all motivational variables for males and females alike.

Lastly, we focused on the impact of self-concept on educational decisions 2 years before students decide upon their courses and basically right after the start of science teaching in Germany. Previous studies have shown that early self-concept already impacts later educational decisions (Nagy et al., 2006; Umarji et al., 2018). For instance, the effects of math and English self-concepts on math intensive college majors were rather similar for the students in the 7th and 10th grades (Umarji et al., 2018). Moreover, there is evidence that motivational profiles are rather stabile from Grades 9 to 10 (e.g., Lazarides et al., 2019). Further, our study did not take self-concept development throughout secondary level into account. On the basis of our results, studies incorporating science and nonscience self-concepts into growth mixture models and using several measurement points in lower secondary education would be suitable to identify subgroups of students that differ in their growth rates of achievement or in their self-concept patterns. One study has already been able to show that students who increased their math self-concept throughout secondary school were more likely to choose a math intensive course than students whose math self-concept declined over the course of secondary school (Musu-Gillette et al., 2015). Those results could be expanded by using a wider array of self-concept variable patterns and by focusing on science course selection, which could then be linked to several outcomes that we used for our study. Thus, future research could thereby provide deeper insights into the substantial changes that might occur in the development of self-concept in different domains and that could influence later science course selection.

## Conclusion

Our study contributes to the body of research on self-concept by incorporating self-concept in several domains and the DCT to understand gendered paths into STEM. To the best of our knowledge, this is the first study that separately tested the self-concept profiles of females and males in upper secondary school in a person-centered and multigroup framework in order to disentangle gendered pathways to academic choices regarding science. Our study further provides evidence for the robustness of former findings regarding multidimensional self-concept profiles using LPA.

## Ethics Statement

Participation in the achievement tests was mandatory for all students with the consent of the Ministry of Schools and Vocational Training of the Free and Hanseatic City of Hamburg, whereas participation in the questionnaires was voluntary. This study was carried out in accordance with the ethical guidelines for research with human participants as proposed by the American Psychological Association (APA). The study materials and procedures were approved by the Ministry of Schools and Vocational Training of the Free and Hanseatic City of Hamburg.

## Author Contributions

SS wrote the first and consecutive drafts of the manuscript. Both the authors listed have made a substantial, direct and intellectual contribution to the work, including the approval for publication.

## Conflict of Interest Statement

The authors declare that the research was conducted in the absence of any commercial or financial relationships that could be construed as a potential conflict of interest.

## Footnotes

1 We also tested models with freely estimated variance. However, these models did not converge, possibly due to an overparameterization (Chen et al., 2001). We therefore used fixed-variance models in all subsequent analyses.
