# Supplementary material for: Self-Concept Profiles in Lower Secondary Level – An Explanation for Gender Differences in Science Course Selection?
Source: Front Psychol. 2019 Apr 24;10:836. doi: 10.3389/fpsyg.2019.00836 (PMC6491640; doi:10.3389/fpsyg.2019.00836)
Supplement: Supplementary file 1 [file Table_1.docx]

Appendix

Table A1. Correlations males

|  |  | 1 | 2 | 3 | 4 | 5 | 6 | 7 | 8 | 9 | 10 | 11 | 12 | 13 |
| --- | --- | --- | --- | --- | --- | --- | --- | --- | --- | --- | --- | --- | --- | --- |
| 1 | MA8 |  |  |  |  |  |  |  |  |  |  |  |  |  |
| 2 | REA8 | 0.45 |  |  |  |  |  |  |  |  |  |  |  |  |
| 3 | SCI8 | 0.44 | 0.41 |  |  |  |  |  |  |  |  |  |  |  |
| 4 | EN8 | 0.39 | 0.39 | 0.28 |  |  |  |  |  |  |  |  |  |  |
| 5 | GMA8 | 0.49 | 0.29 | 0.40 | 0.17 |  |  |  |  |  |  |  |  |  |
| 6 | GGER8 | 0.29 | 0.33 | 0.26 | 0.22 | 0.52 |  |  |  |  |  |  |  |  |
| 7 | GBIO8 | 0.27 | 0.28 | 0.33 | 0.1 | 0.52 | 0.48 |  |  |  |  |  |  |  |
| 8 | GEN8 | 0.31 | 0.30 | 0.22 | 0.48 | 0.40 | 0.51 | 0.38 |  |  |  |  |  |  |
| 9 | GPHY8 | 0.37 | 0.26 | 0.36 | 0.11 | 0.62 | 0.43 | 0.51 | 0.37 |  |  |  |  |  |
| 10 | SC-BIO | 0.09 | 0.12 | 0.18 | -0.04 | 0.21 | 0.10 | 0.45 | 0.03 | 0.23 |  |  |  |  |
| 11 | SC-PHY | 0.23 | 0.13 | 0.28 | -0.06 | 0.42 | 0.12 | 0.30 | 0.09 | 0.54 | 0.31 |  |  |  |
| 12 | SC-REA | 0.13 | 0.21 | 0.22 | 0.25 | 0.08 | 0.17 | 0.06 | 0.18 | 0.06 | 0.12 | 0.10 |  |  |
| 13 | SC-MA | 0.41 | 0.09 | 0.28 | 0.04 | 0.6 | 0.13 | 0.27 | 0.13 | 0.42 | 0.21 | 0.49 | 0.05 |  |
| 14 | SC-EN | 0.08 | 0.16 | 0.02 | 0.46 | -0.03 | 0.14 | 0.06 | 0.45 | -0.01 | -0.01 | -0.09 | 0.29 | -0.07 |

*Note.* MA8 = math test score; REA8 = reading test score; EN8 = English test score; GMA8 = grade in math; GGER8 = grade in German; GBIO8 = grade in biology; GEN8 = grade in English; GPHY8 = grade in physics; SC-BIO = self-concept in biology; SC-PHY = self-concept in physics; SC-REA = self-concept in reading; SC-MA = self-concept in math; SC-EN = self-concept in English

Table A2. Correlations females

|  |  | 1 | 2 | 3 | 4 | 5 | 6 | 7 | 8 | 9 | 10 | 11 | 12 | 13 |
| --- | --- | --- | --- | --- | --- | --- | --- | --- | --- | --- | --- | --- | --- | --- |
| 1 | MA8 |  |  |  |  |  |  |  |  |  |  |  |  |  |
| 2 | REA8 | 0.43 |  |  |  |  |  |  |  |  |  |  |  |  |
| 3 | SCI8 | 0.46 | 0.42 |  |  |  |  |  |  |  |  |  |  |  |
| 4 | EN8 | 0.43 | 0.41 | 0.35 |  |  |  |  |  |  |  |  |  |  |
| 5 | GMA8 | 0.47 | 0.26 | 0.34 | 0.29 |  |  |  |  |  |  |  |  |  |
| 6 | GGER8 | 0.31 | 0.35 | 0.25 | 0.3 | 0.48 |  |  |  |  |  |  |  |  |
| 7 | GBIO8 | 0.31 | 0.27 | 0.29 | 0.23 | 0.50 | 0.46 |  |  |  |  |  |  |  |
| 8 | GEN8 | 0.37 | 0.29 | 0.28 | 0.48 | 0.47 | 0.52 | 0.42 |  |  |  |  |  |  |
| 9 | GPHY8 | 0.40 | 0.28 | 0.30 | 0.24 | 0.60 | 0.46 | 0.52 | 0.44 |  |  |  |  |  |
| 10 | SC-BIO | 0.11 | 0.15 | 0.21 | 0.05 | 0.17 | 0.17 | 0.49 | 0.11 | 0.26 |  |  |  |  |
| 11 | SC-PHY | 0.26 | 0.17 | 0.28 | 0.05 | 0.37 | 0.19 | 0.32 | 0.16 | 0.58 | 0.32 |  |  |  |
| 12 | SC-REA | 0.17 | 0.35 | 0.23 | 0.25 | 0.06 | 0.29 | 0.16 | 0.21 | 0.14 | 0.21 | 0.12 |  |  |
| 13 | SC-MA | 0.47 | 0.16 | 0.28 | 0.12 | 0.65 | 0.21 | 0.27 | 0.24 | 0.47 | 0.17 | 0.45 | 0.03 |  |
| 14 | SC-EN | 0.15 | 0.18 | 0.10 | 0.44 | 0.09 | 0.22 | 0.13 | 0.50 | 0.11 | 0.05 | 0.01 | 0.35 | -0.04 |

*Note.* MA8 = math test score; REA8 = reading test score; EN8 = English test score; GMA8 = grade in math; GGER8 = grade in German; GBIO8 = grade in biology; GEN8 = grade in English; GPHY8 = grade in physics; SC-BIO = self-concept in biology; SC-PHY = self-concept in physics; SC-REA = self-concept in reading; SC-MA = self-concept in math; SC-EN = self-concept in English


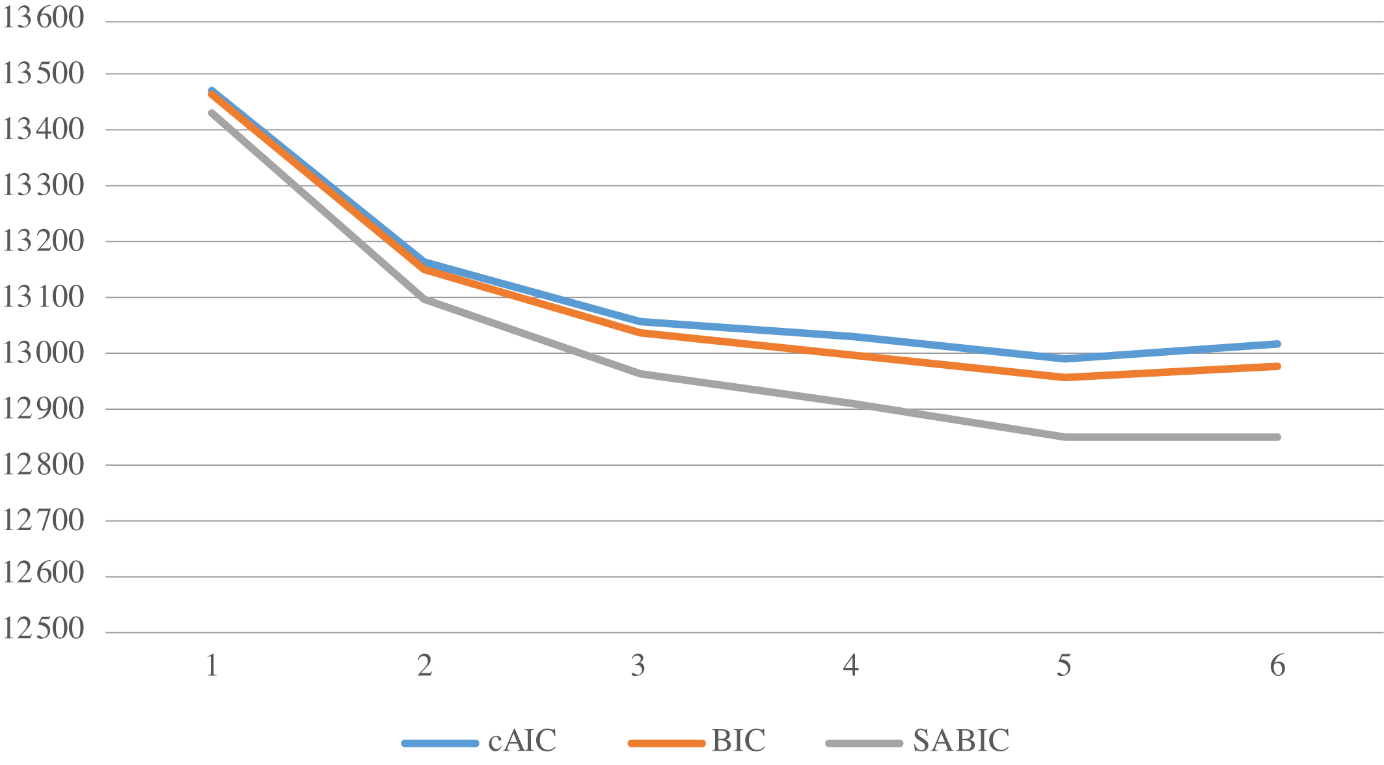


FIGURE A1. Elbow plots for the LPA (females)


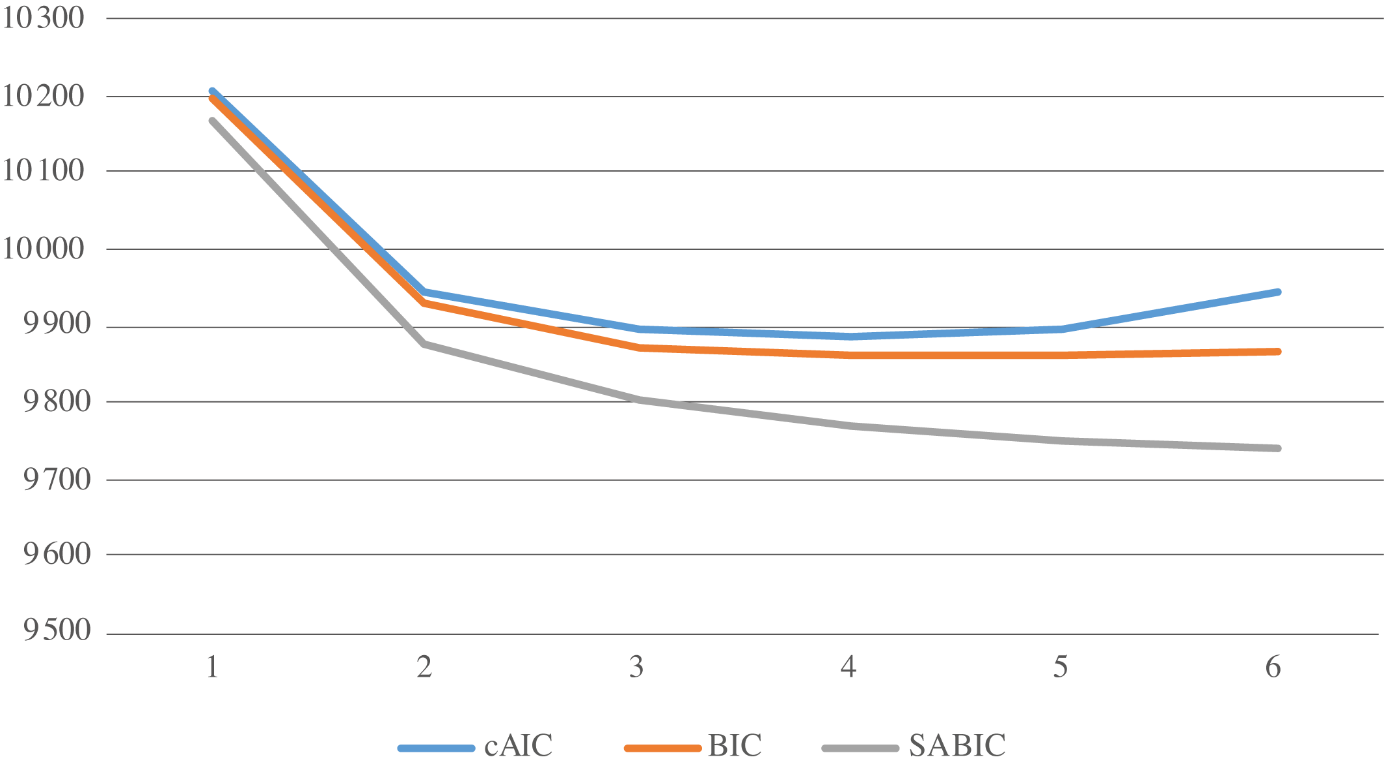


FIGURE A2. Elbow plots for the LPA (males)

TABLE A3. Chi-squared test for gender differences

|  | HMRSC |  | LOSC |  | HVRSC |  | HOSC |  |
| --- | --- | --- | --- | --- | --- | --- | --- | --- |
|  | females | males | females | males | females | males | females | males |
| observed | 60 | 129 | 160 | 68 | 460 | 204 | 264 | 313 |
| ASR | -7.4 | 7.4 | 4.3 | -4.3 | 8.3 | -8.3 | -6.7 | 6.7 |

Note. Overall χ2 = 21.67, df=3, p < .001; ASR stands for adjusted standardized residuals.


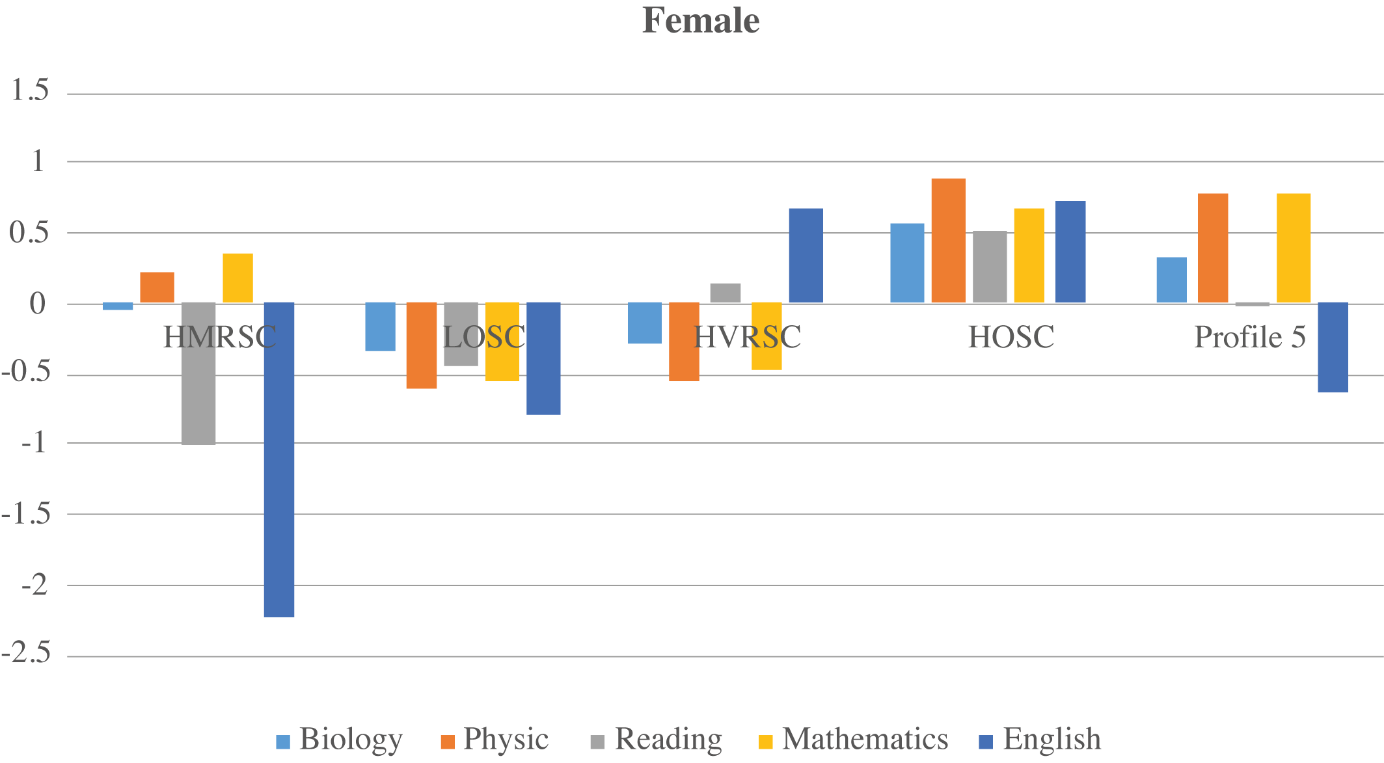


FIGURE A3. 5-profile solution LPA (females)


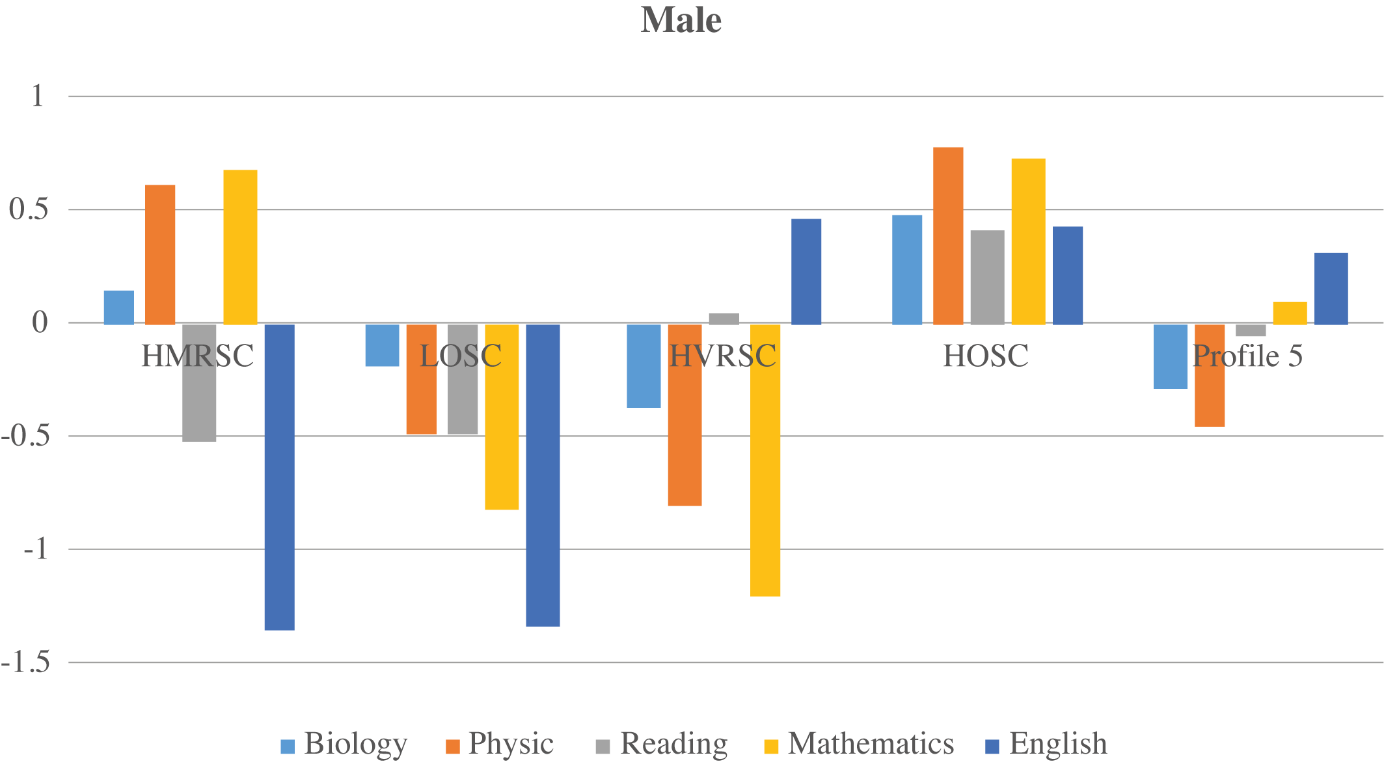


FIGURE A4. 5-profile solution LPA (males)
